# Bacterial Urease and its Role in Long-Lasting Human Diseases

**DOI:** 10.2174/138920312804871094

**Published:** 2012-12

**Authors:** Iwona Konieczna, Paulina Żarnowiec, Marek Kwinkowski, Beata Kolesińska, Justyna Frączyk, Zbigniew Kamiński, Wiesław Kaca

**Affiliations:** 1 Department of Microbiology, Institute of Biology, The Jan Kochanowski University, ul. Świętokrzyska 15, 25-406 Kielce, Poland; 2 Technical University of Lodz, Institute of Organic Chemistry, ul. Żeromskiego 116, 90-924 Łódź, Poland.

**Keywords:** Antibodies, long-lasting diseases, synthetic peptides, urease

## Abstract

Urease is a virulence factor found in various pathogenic bacteria. It is essential in colonization of a host organism and in maintenance of bacterial cells in tissues. Due to its enzymatic activity, urease has a toxic effect on human cells. The presence of ureolytic activity is an important marker of a number of bacterial infections. Urease is also an immunogenic protein and is recognized by antibodies present in human sera. The presence of such antibodies is connected with progress of several long-lasting diseases, like rheumatoid arthritis, atherosclerosis or urinary tract infections. In bacterial ureases, motives with a sequence and/or structure similar to human proteins may occur. This phenomenon, known as molecular mimicry, leads to the appearance of autoantibodies, which take part in host molecules destruction. Detection of antibodies-binding motives (epitopes) in bacterial proteins is a complex process. However, organic chemistry tools, such as synthetic peptide libraries, are helpful in both, epitope mapping as well as in serologic investigations.

In this review, we present a synthetic report on a molecular organization of bacterial ureases - genetic as well as structural. We characterize methods used in detecting urease and ureolytic activity, including techniques applied in disease diagnostic processes and in chemical synthesis of urease epitopes. The review also provides a summary of knowledge about a toxic effect of bacterial ureases on human body and about occurrence of anti-urease antibodies in long-lasting diseases.

## 1 INTRODUCTION 

Urease (urea amidohydrolase; EC 3.5.1.5) was the first enzyme to be crystallized (1926). It was also the first enzymatic protein in which the presence of nickel ions was noted [1]. Since then, an intensive study on urease has been conducted, thanks to which a role of urease in nitrogen compounds circulation has been determined. It has also been showed that urease may be a virulence factor essential in various illnesses, including long-lasting diseases. 

**Fig. (1):**
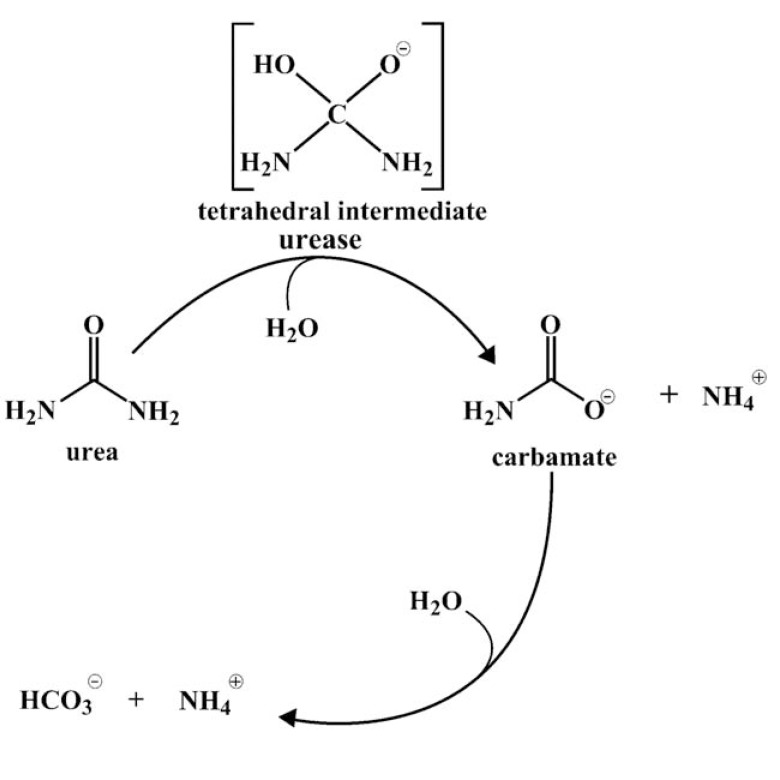
Scheme of urea hydrolysis.

Urease is capable of urea hydrolysis. This compound is widespread: it is found in the natural environment (water and soil) and in human body, where its occurrence is connected with protein degradation. In humans, urea is a factor of normal functions of kidneys [2, 3]. A healthy adult excretes about 30 g of urea per day [2]. However, it is present not only in urine, but also in blood serum, sweat and even in stomach [1, 2]. Hydrolysis of urea by urease is a complex process. In the first step, one molecule of ammonia and one molecule of carbamate appear. In water solution, carbamate spontaneously converts into the second ammonia molecule and carbonic acid. Next ammonia is protonated (Fig. **1**). This process results in pH increase [1]. 

Urease and ammonia, generated during urea hydrolysis, may be toxic for human tissue [4, 5] and probably have role in long-lasting diseases, like atherosclerosis or rheumatoid arthritis [6, 7]. This phenomenon will be precisely described in the next paragraphs. 

## 2 OCCURRENCE OF UREASE PRODUCING ORGANISMS

Urease is produced by many different bacteria [8-17], fungi [3, 18, 19], plants [1, 3, 8, 20, 21] and even some invertebrates [20, 21]. Microorganisms with ureolytic properties were found in soil and water as well as in human and animal bodies [8]. Ureolytic bacteria may belong to symbiotic natural microflora or to pathogens. In facultative anaerobes from intestinal microflora the level of this activity is diverse and species characteristic [9].

Ureolytic activity is often observed in pathogenic bacteria. Such a feature is characteristic of pathogenic *Staphylococcus* strains. Over 90% of clinical methicillin resistant *Staphylococcus aureus* strains are capable of urea hydrolysis [10]. *Staphylococcus leei* isolated from biopsy material from gastritis patients was also ureolytic. Uropathogenic *Staphylococcus saprophiticus* is also capable of this activity [1, 11, 22]. Urease is observed in *Helicobacter* sp., including all *Helicobacter pylori* isolated from gastritis patients [1, 4, 23]. Urease is an enzyme synthesized by pathogenic mycobacteria like *Mycobacterium tuberculosis* and *Mycobacterium bovis* [12]. It was observed that anaerobic clostridia are capable of urea hydrolysis. About 2% of *Clostridium perfringers* strains, an etiologic factor of gas gangrene, showed this feature [13]. Even some strains of *Vibrio parahaemolyticus*, a species considered non-ureolytic, produce urease [14]. Another generally urease negative bacterial species is *Escherichia coli*. Among *E. coli* strains, about 1% of urease-positive isolates were found. This feature was connected with pathogenic O111, O157:H7, O145 and O26 enterohemorrhagic *E. coli*, and in O157 serogroup with sorbitol fermenting, but non motile strains [15-17]. *Proteus mirabilis* is a well-known ureolytic human’s pathogen. Urease is one of the major bacterial virulence factors during urinary tract infections caused by these bacteria [1, 24]. A similar phenomenon was noted for uropathogenic *Ureapasma urealyticum*, *Klebsiella* spp., *Pseudomonas* spp., *Corynebacterium* sp. D2, *Proteus penneri*, *Providencia stuartii* and *Morganella morganii* [1, 22]. 

## 3 GENETIC AND STRUCTURAL ORGANIZATION OF BACTERIAL UREASES

Urease is a nickel-containing enzyme, which requires activity of a few additional proteins for acquisition of its hydrolytic properties. This process involves genes coding structural enzyme polypeptides as well as genes coding accessory proteins, located in a joint cluster [1, 25]. 

**Fig. (2):**
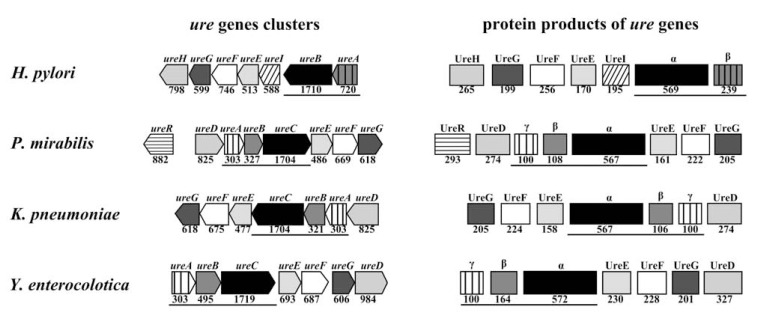
Scheme of genetic organization of urease genes and structural composition of urease. Genetic organization of *ure* genes was performed basing on Microbial Genome Viever MGV 2.0 (http://mgv2.cmbi.ru.nl) for *H. pylori* G27, *P. mirabilis* HI4320, *K. pneumonia* 342 and *Yersinia enterocolitica* 8081. Under genes/polypeptides are sizes of particular genes as well as a number of amino acids of particular polypeptide were taken from NCBI database for records: CP001173 (*H. pylori* G27), AM942759 (*P. mirabilis* HI4320), NC_011283 (*K. pneumonia* 342) and NC_008800 (*Y. enterocolitica* 8081); structural genes as well as urease subunits are underlined.

Bacterial ureases are always multimeric enzymes composed of two or three different polypeptides [1]. In *P. mirabilis,* three structural subunits: 11 kDa UreA (subunit γ), 12.2 kDa UreB (subunit β) and 61 kDa UreC (subunit α) are found [1, 26, 27]. These polypeptides are encoded by three structural genes: *ureA*, *ureB* and *ureC* respectively [28]. Such organization is characteristic of most pathogenic and environmental bacteria. Unique urease of *Helicobacter* sp. has a different structure. In *H. pylori*, urease consists of only two subunits: 26.5 kDa UreA (subunit β) and 61.7 kDa UreB (subunit α) coded by *ureA* and *ureB* genes [29]. A smaller *Helicobacter* sp. urease structural gene (*ureA*) corresponds with a hypothetical fusion gene arisen from *ureA* and *ureB* typical of other bacteria, while a larger gene (*ureB*) is analogous to *ureC* (Fig. **2**) [30-33]. 

Urease composed of two different polypeptides (21 kDa and 65 kDa) was also identified in SL100 ureolytic coccoid strain isolated from stomach biopsy material. This strain was related to *Staphylococcus cohnii* and *Staphylococcus xylosus*, which possess three urease subunits [34].

An active center of enzyme with two metal ions is located in the largest of structural subunits. In all ureases it is designed as UreC, except *Helicobacter* sp., in which case it is UreB [1]. Ureases are nickel-containing enzymes; however, for microaerophilic *Helicobacter mustelae* an iron-containing urease was revealed [23].

All bacterial ureases occur as inactive apoenzymes composed of three or two types of polypeptides coded by specific structural genes. However, additional proteins, products of accessory genes are required for urease activation. Those proteins (UreD, UreE, UreF, UreG and UreH) are involved in transporting nickel ions into a cell and in incorporating them into an active center of apoenzyme [35-44]. *P. mirabilis* produce active urease in presence of urea. In these bacteria a regulatory gene *ureR* is present (see Fig. **2**). Its product is a urea inducible regulator controlling expression of remaining genes [1].

A highly mobile helix-turn-helix motif, located in α subunit and called “flap” is essential for urease activity (see Fig. **5**). It may adopt two different conformations. In the “open” position, urea may enter into the active site, where hydrolyze is performed. In the “closed” position, flap covers the active center and blocks access to it [25].

Active ureases are heterooligomeric complexes. However, the number of particular structural subunits is always equal. In *K. aerogenes* urease, as well as in other tree-subunit bacterial ureases, UreC/UreB/UreA molecules occur in the ratio 1:1:1. Likewise, for *Helicobacter* sp. UreB/UreA are always in the ratio 1:1 [1]. 

**Fig. (3):**
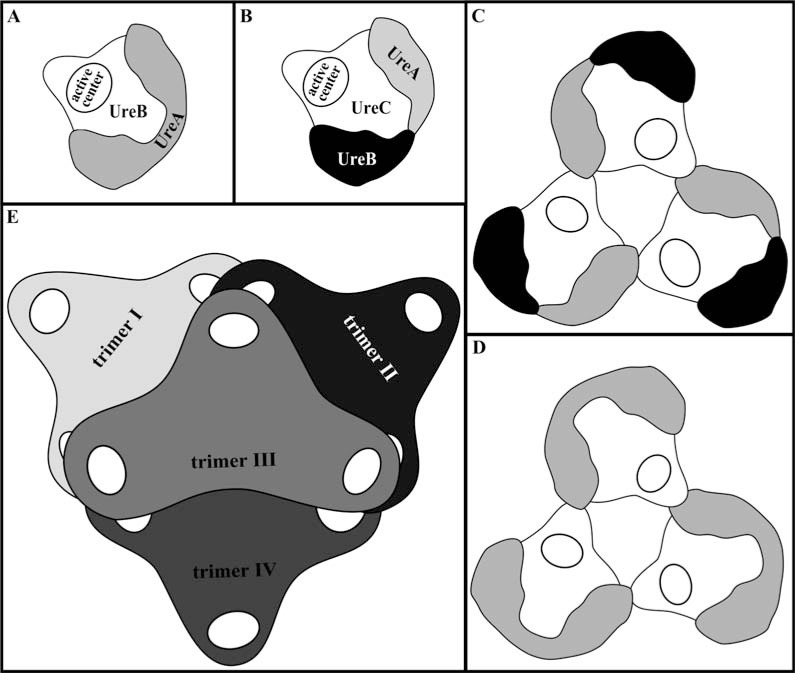
Scheme of structure of bacterial ureases. Domain organization is reported for (A) *H. pylori* and (B) *Bacillus pasteurii* urease monomers, (C) *B. pasteurii* and (D) *H. pylori* urease trimers, and (E) *H. pylori* dodecamer.

Urease from *K. aerogenes*, as well as the most of other bacteria, is triple trimer (αβγ)_3_ with three active centers, one in each of three α subunits. Amino- and carboxyl terminus of each subunit are free and they are able to bind additional compounds without disturbing the enzyme structure [1]. But *Prochrorococcus marinus *sp. PCC 9511 produces urease composed according to (αβγ)_2_ pattern [1, 45]. Enzymes from *Helicobacter* sp. may form a more complex structure, built from 12 subunits. Polypeptides α and β are linked forming trimer (αβ)_3_, where N-terminal domain of β subunit are essential in aggregation process. Then, four such trimers form a tetrahedral complex (Fig. **3**). 

Probably, such a highly complex structure of *H. pylori* urease enables its activity in acidic conditions, when other ureases undergo nonreversible inactivation [46].

## 4 CONSERVATISM OF BACTERIAL UREASES

Ureases are considered to be conservative enzymes. Among all Ure polypeptides from different bacterial species, the highest sequence similarities were observed between structural urease subunits from different sources. In case of remaining polypeptides, similarities were smaller (Table **1**.) [47, 48].

**Fig. (4):**
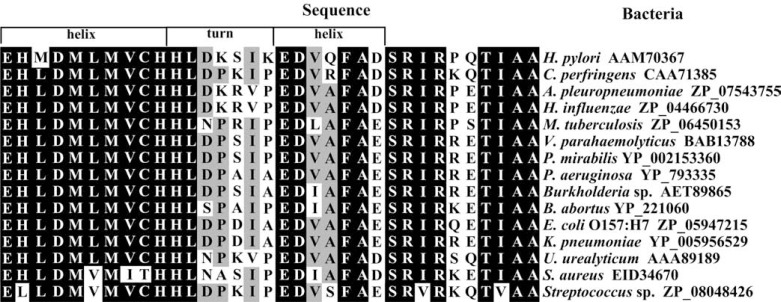
Alignment of the amino acid sequence of urease flap fragment from pathogenic bacteria. Black - amino acid present in at least 80% of compared sequences, grey - amino acid present in at least 70% of compared sequences, white - amino acid present in less than 70% of compared sequences. Sequences from NCBI database (Accession Numbers are in figure). Alignment was performed by Clustal W 2.1 and edited with GeneDoc.

In α subunit, the active center is the most conservative. Particularly stable are nickel ligands: histidines (in *K. aerogenes* His-134, His-136, His-246 and His-272), lysine (in *K. aerogenes* Lys-217) and aspartic acid (in *K. aerogenes* Asp-360) [1, 51]. Similarities were also observed for the flap fragment from over 160 sequences of α subunit of ureases from different microorganisms, including human pathogens. Many sequential identities occurred in all amino acid sequences. In bacteria causing human diseases, even not closely related, significant conservatism was noted (Fig. **4**) [52].

**Fig. (5):**
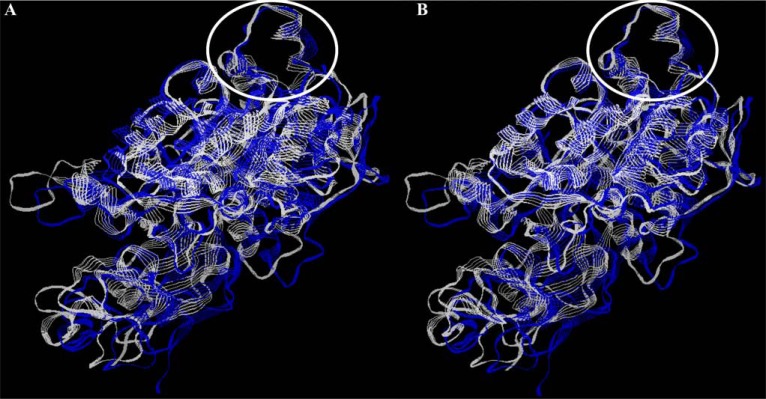
Conformational conservatism of bacterial ureases. All models of bacterial ureases were from ExPASy SIB Bioinformatics Resource Portal (Q7X3W5 - *H. pylori*; P16122 - *P. hauserii*; Q6GEE4 - *S. aureus*); overlapping was performed with RasWin Molecular Graphics Visualisation Tool (http://rasmol.org/). A - structure of *H. pylori* (blue) and *P. hauserii* (white) ureases, B - structure of *H. pylori* (blue) and *S. aureus* (white) ureases; flap region is marked by a white ellipse.

The structure of a flap region in ureases from different bacteria also possess similar conformation (Fig. **5**). Bacterial pathogens shows different ureolytic activity. Methods for its detection, including techniques applied in disease diagnosis, are diverse.

## 5 METHODS FOR DETECTING OF UREOLYTIC ACTIVITY

Hydrolysis of urea is one of useful features in the bacteria identification. In a few infections caused by microbes, detection of this activity is essential to disease diagnosis. 

Numerous assays are available to determine urease activity as well as to analyze kinetic behavior of urease. Most of them are indirect and based on colorimetric detection of ammonia released during incubation with a buffered urea solution [53]. 

One of the first methods was detecting bacterial ureolytic activity based on the cultivation of microorganism on urea containing medium (Christensen's urea medium) [54]. This is the most popular qualitative method using for uropathogenes like *Proteus* sp. In case of this bacterial species, results may be obtained even after 4 h. A modification of Christensen technique allows reducing assay time [54, 55].

Ureolytic activity is one of biomarkers employed to diagnose *H. pylori* infection and to monitor bacteria eradication by drug treatment [56].

For diagnosis, invasive and noninvasive tests, depending on whether endoscopy is required or not, are applied. The most popular invasive test is a rapid urease test (RUT) that requires obtaining tissue samples. However, this method is inconvenient for patients and also incurs high costs [57, 58]. It requires biopsy specimens from defined regions of the stomach. This material is placed on a urea-containing medium. If bacteria are present in the specimen, the change of color resulted from alkalization of the medium is observed [59]. A urea breath test (UBT) is commonly used among noninvasive tests. This method is simple, but its performance may be slightly complicated in case of very young children as well as patients with certain neurological disorders [49, 50 60]. It involves oral administration of a nontoxic isotopically labeled (C^14^ or C^13^) urea to a patient. Urea is hydrolyzed by *H. pylori* to ammonia and isotope-containing CO_2_. Carbon dioxide is dissolved into blood and removed via lungs. Isotopes are detected in exhaled air. This is a test of choice in medical practice for detecting *H. pylori* infection. [61]. There are also suggestions that a urea breath test may be applied for diagnosing tuberculosis [62].

Other methods are used mainly in scientific research. Each of the methods developed for determination of ureolytic activity has some advantages and disadvantages (Table **2**).

There are a lot of other methods based on detecting ammonia released by urease action, which can be determined by vacuum distillation, a microdiffusion, steam distillation and electroconductivity measurement [77]. 

**Fig. (6):**
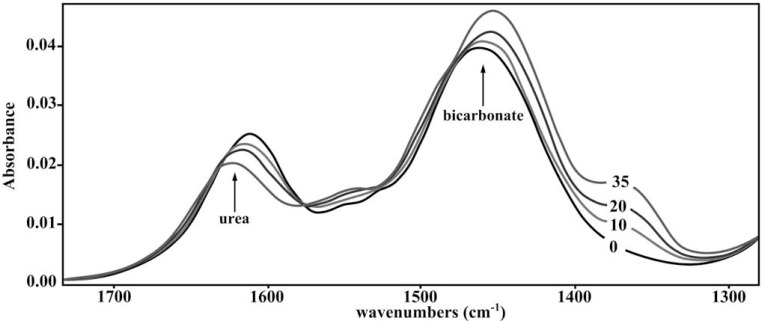
ATR FTIR spectra of the substrate (urea), product (NaHCO_3_), in reaction mixture containing 0.4 µg urease from *Canavalia ensiformis*. Spectra of the reaction mixture were recorded at several time intervals: 0, 10, 20 and 35 min, as indicated.

Fourier Transform Infrared (FTIR) spectroscopy is a method raising a big hope for easy, quick and continuous detection of ureolytic activity. This technique constitutes a radically different approach to enzymatic activity determination. It is based on the measurement of molecular vibrations energy of functional groups in organic compounds. This makes FTIR spectroscopy a highly sensitive and reproducible method. Unlike the previously discussed methods, this technique enables continuous monitoring of enzymatic reaction by a simultaneous analysis of disappearance of substrate and the appearance of product. However, substrate as well as product must have different spectra. FTIR spectroscopy also enables enzyme kinetics investigation [78]. Attenuated Total Reflection Fourier Transform Infrared (ATR-FTIR) spectroscopy was recommended to enzymatic activity analysis [79]. This technique was also applied for urease activity investigations. Bands of absorbance characteristic of urea (substrate) and of bicarbonate (product) could be easily monitored in time intervals (Fig. **6**). [Zarnowiec *et al.*, data unpublished]. However, ATR-FTIR spectroscopy is now used only in research applications, not in routine medical practice. 

## 6 UREASE AS A PATHOGENIC BACTERIA VIRULENCE FACTOR

Bacterial ureases play a role in disease pathogenesis. They are connected with urinary stones occurrence and catheters blocking, pyelonephritis, ammonia encephalopathy, hepatic coma as well as gastritis. In many papers there are information concerning toxic effects of bacterial ureases (Table **3**).

The role of urease in bacterium surviving in unfavorable microenvironment in the host’s body is especially noticeable in case of *H. pylori*, a causative agent of gastritis and peptic ulceration [1, 4]. At *in vitro* conditions, *H. pylori* is sensitive to low pH. During infection, microorganisms have to pass through gastric acid before reaching the protective mucus layer. In these circumstances, a pathogen produces a large amount of urease which is not observed in other bacteria [80]. At low pH, enzymatic activity of *H. pylori* urease is probably connected with its dodecameric structure. This enzyme is also able to perform a more efficient hydrolysis of urea. This property may be due to mobility of the flap region, which is different than in *K. aerogenes* or *B. pasteurii* ureases [46]. Due to the high activity of *H. pylori* urease, local microenvironment surrounding bacterium becomes nearly neutral. Moreover, live bacterial cells adsorb on the surface enzymes released upon other *H. pylori* autolysis, which makes it possible for them to get to gastric mucus layer safely [80]. Ureolytic activity is essential for surviving* M. tuberculosis*, an etiologic factor of tuberculosis, a long-lasting inflammatory lung disease. Bacteria infect macrophages. They reside in phagosome, where alkalization due to ureolytic activity and subvert phagosome maturation takes place. Additionally, urease activity enables bacterium to exist in the environment where nitrogen sources are limited to urea [81]. Ureolytic activity is useful in better surviving of bacteria also in case of uropathogenes. Urease facilitates urinary tract infection. Infection dose for ureolytic *P. mirabilis* HI4320 was 1000-times lower in comparison with its non-ureolytic mutant. Urease activity raises pH of human urine, which allows precipitation of normally soluble polyvalent ions to struvite and carbonate apatite. These compounds aggregate around bacteria, forming urinary stones. Inside such stones, microorganisms are protected from antibiotics and the host’s immune system [24, 83]. Urinary stones block urethra or catheters leading to acute bacteriuria [24]. The role of ureolytic activity in urinary stones formation was also showed for *U. urealyticum*, *S. saprophiticus*, *S. aureus* and some *Klebsiella* spp., *Pseudomonas* spp., as well as *Corynebacterium* sp. D2, *P. penneri*, *P. stuartii*, *M. morganii* [1, 22]. 

One of the features essential in bacterial infections is persistence to the host’s cells. Schoep *et al.* showed that *H. pylori* urease have two sites (one at the N-termini of UreA subunit and the other at C-termini of UreB) which were involved in persistence to endothelial cells during mouse colonization [29]. This observation was confirmed by investigations with urease-negative *H. pylori* mutants incapable of colonization [1,4]. Moreover, also urease released from lyzed bacterial cells is capable of adsorption into the mucus layer [4].

Bacterial ureases affect host immune system cells. In *H. pylori* infection, this metalloenzyme activates monocytes and neutrophils, which leads to secretion of inflammatory cytokines and causes indirect damage to epithelial cells. Urease is a chemotactic factor for monocytes and neutrophils. Inflammatory reaction may also be initiated by adsorption of released enzyme into the mucus layer [4]. Induction of inflammatory reaction was also observed for *Y. enterocolitica* urease. Ability of bacterial UreB subunit to induce experimental reactive arthritis was revealed [1, 84]. 

Urease may contribute in damaging host’s cells. Enzyme from *H. pylori* stimulates expression of inducible NO-synthesizing enzyme (iNOS), which may have a cytotoxic effect [80]. Urease may exert a toxic effect also indirectly, by ammonia - the product of urea hydrolysis. During *H. pylori* infection, a stimulation of an oxidative burst in neutrophils ensues and there is a release of hydrogen peroxide, which next oxidizes chlorine ions. Ammonia generated by urease reacts with them and gives toxic monochloramine [1]. Johnson *et al.* revealed, using mouse model, that ammonia causes tissue damage also during urinary tract infections with ureolytic *P. mirabilis*. In kidneys, an acute inflammation as well as necrotic cells were observed. After one week, pyelonephritis was in progress. Struvite stones were noted. After two weeks, kidneys were ulcerated and fibrosis was visible [87]. Moreover, ammonia released by urease causes damage to the glycosaminoglycan layer in urothelial surface, and disturbs its protective function [5].

Recently, a new role of *H. pylori* urease has been established. During an infection, bacteria cause increased phosphorylation of the myosin regulatory light chain. Such phosphorylation regulates the function of epithelial tight junction complexes, which have a role in maintenance of barrier function, cell polarity as well as intercellular adhesion. Disruption of tight junction is associated with a carcinogenesis process. Wroblewski *et al.* showed that *H. pylori* urease may be connected with gastric cancer by causing damage to tight junctions [85].

Lately, a mechanism of activating blood platelets by bacterial urease has been described. Wassermann *et al.* showed that *H. pylori* enzyme stimulates this process through a lipoxygenase-mediated pathway. Such properties may have a role not only in gastrointestinal, but also cardiovascular diseases [86]. 

## 7 PRESENCE OF ANTI-UREASE ANTIBODIES IN SERA OF PATIENTS WITH LONG-LASTING DISEASES

Bacterial ureases are considered to be one of the major antigens in several human diseases [1, 83, 84, 88]. Hirota *et al.* showed that this protein is immunogenic [89]. In the flap region of enzyme, the ELR motive associated with immunogenic antigens is present (see Fig. **4**) [90]. In long-lasting diseases caused by ureolytic bacteria, urease may stimulate generation of antibodies.

Infections of *H. pylori* are mostly chronic and, in many cases, lifelong [91]. During a infection, an elevated level of immunoglobulins (secretory as well as circulating) was observed [88]. Different classes of antibodies were noted: in the stomach - IgA and IgM, in serum - IgG and IgA. IgG immunoglobulins remain even for a few months after bacterium eradication [92]. Urease from this bacterium is one of the major immunodominant antigens [93]. It is considered a vaccine in preventing *H. pylori* infections. In animal model, vaccination with *H. pylori* urease provides a significant and long term protection against a bacterial infection. In humans, oral administration of such a vaccine resulted in a strong immune response with minimal side effects [88].

The presence of anti-urease antibodies in *H. pylori* seropositive individuals is correlated with age and living in highly developed regions. Leal-Herrera, in the investigations performed on a population in Mexico, revealed that the percentage of infected individuals increases with age. The presence of anti-urease IgG antibodies in serum rises from less than 20% in a group of individuals below 10 years old to more than 50% - in a group over 40 years old [93]. Occurrence of anti-urease antibodies was correlated with disease severity. In patients with superficial gastritis, a low level of IgG, but relatively high of IgA immunoglobulins was observed. Strong IgG reaction dominated in quiescent atrophic gastritis individuals, whereas in patients with active atrophic gastritis, reaction of IgG as well as IgA was very strong [15].

**Fig. (7):**
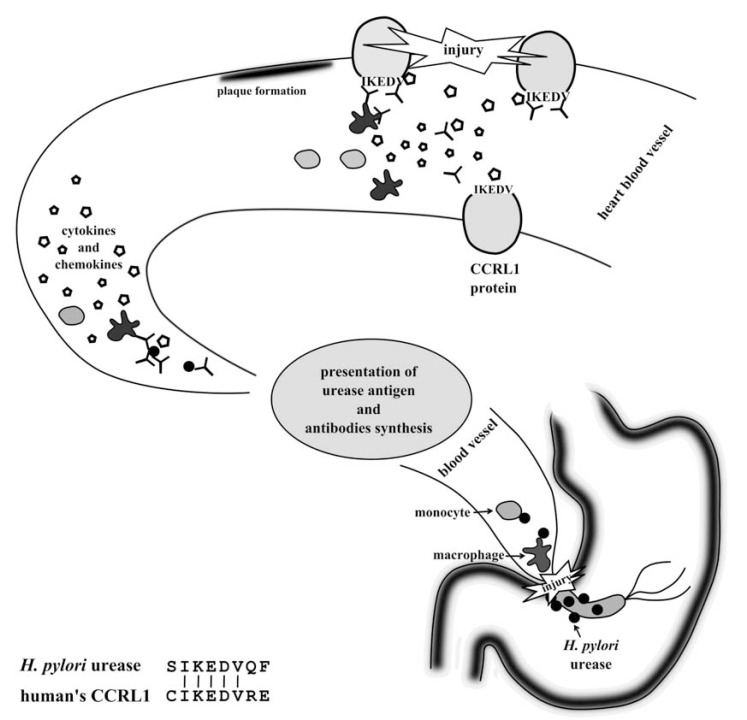
Molecular mimicry of flap fragment of *H. pylori* urease and CCRL1 and possible connection with atherosclerosis progress.

Nurgalieva *et al.* observed the presence of IgM antibodies, putatively recognizing a small subunit (UreA) of *H. pylori* urease in 94% of *H. pylori*-infected volunteers. The larger subunit - UreB seems to be less immunogenic. About 44% of the investigated individuals showed a positive reaction [94]. However, Burnie and Al-Dughaym showed that UreB subunit of *H. pylori* urease has more epitopes recognized by antibodies than UreA. The level of IgG antibodies recognizing some of those epitopes was comparable with the commercial test [95]. Also in Arabski *et al.* study, where levels of IgG antibodies were detected, the presence of antibodies recognizing *H. pylori* UreB urease subunit was found in almost all infected individuals. They were observed even in 70% of *H. pylori* negative sera. A much more interesting observation was a correlation between atherosclerosis and the presence of anti-urease antibodies. In the investigated sera there was a significant relationship between the level of antibodies bound to 8-mer synthetic peptide (which corresponds to UreB minimal flap epitope of *H. pylori* urease) and occurrence of atherosclerosis [6], an inflammatory disease leading to an atheromatosus plaque in blood vessels lumen [96]. Earlier, Oshima *et al.* suggested that chronic *H. pylori* infections are connected with inflammatory processes in vessels [97]. Investigations applying synthetic peptide corresponding to 8 amino acid sequence of flap fragment of *H. pylori* urease revealed a similarity between this peptide and human CCRL1 (CC chemokine receptor-like 1) protein, expressed mainly in the heart. Based on this observation, a hypothesis to explain a connection of *H. pylori* urease and atherosclerosis was formulated. According to it, urease may stimulate immune system reaction during a bacterial infection. Presentation of urease fragments to Th lymphocytes enables synthesis of antibodies. Next, antibodies against flap region of urease react with bacterial antigen. However, they may also recognize IKEDV motive in CCRL1 (due molecular mimicry) and cause an inflammatory process (Fig. **7**) [6]. 

Rheumatoid arthritis (RA) is a classic long-lasting disease. It is an inflammatory condition leading to joint injury. During its progress, hyaline cartilages of joints as well as bones undergo atrophy [98]. Etiology of RA is complex and, despite many years of investigations, still unclear. Apart from genetic background of RA occurrence, a role of infectious agents, like *P. mirabilis*, *Borrelia burgdorferi*, *Mycoplasma* sp., *M. tuberculosis*, *E. coli*, and *Porphyromonas gingivalis* as well as some viruses was discussed [99, 100]. Some of them are capable of urease synthesis [12, 24]. Among them, the most important is *P. mirabilis*. Wilson *et al.* revealed a connection of bacterial urease with disease progress. They showed a molecular mimicry between IRRET motive in *P. mirabilis* urease and human type XI collagen (LRREI sequence) present in hyaline cartilage. The observed similarities concerned a sequence as well as a conformation fragments of both proteins. Simultaneously, the level of antibodies against *P. mirabilis* urease was significantly higher in comparison to healthy individuals as well as patients with ankylosing spondylitis - another autoimmune disease. According to Wilson *et al.* hypothesis, antibodies arising in reaction against bacterial urease function as autoantibodies and recognize also human protein (collagen). This leads to primary cytotoxic damage to hyaline cartilage. In the next step, in an injury site the presence of cytokines, vascular adhesion molecules and hydrolytic enzymes is observed. It causes inflammation, fibrosis and destruction of joints [101].

This hypothesis was confirmed in later studies. Konieczna *et al.* observed for RA patient’s sera a significantly higher level of antibodies recognizing synthetic peptide corresponding to flap epitope of *P. mirabilis* urease. Surprisingly, they noted an elevated IgG level against peptides reflecting a sequence of flap regions from other organisms (bacteria and plant). The detected antibodies also had lower specificity. These antibodies recognized not only one defined antigen, but also antigens with a similar sequence, which was probably due to instability of the immune system [7]. 

A role of urease in stimulation of immune response of patients with immune disease was also revealed for other gram-negative bacteria. In 1993, it was showed that β subunit of *Y. enterocilitica* O:3 urease is arthritogenic for rats [84]. A few years later, a high humoral response in patients with reactive arthritis triggered by *Y. enterocolitica* O:3 was noted. IgG reacting with 19 kDa urease subunit was observed in over 90%, and IgA in over 50 % of investigated sera [102].

In chronic obstructive pulmonary disease (COPD) caused by nonencapsulated *H. influenza*e, urease is a target of human humoral response. In almost 39% of investigated sera, a significantly higher level of antibodies reacting with bacterial urease was observed [103].

Anti-urease antibodies are detected even in case of chronic zoonosis. In patients with diagnosed brucellosis, antibodies recognizing α *Brucella suis* urease subunit were detected [104].

In the investigations of antibodies generated as a response to infection, chemically defined synthetic peptides have a potent application. They are useful for epitope mapping as well as molecular mimicry studies.

## 8 ORGANIC CHEMISTRY TOOLS IN IMMUNE RESPONSE INVESTIGATIONS

Organic chemistry enables constructing several new tools for investigations of response of the immune system against infectious agents. One of these tools is a library of synthetic peptides with a chemically defined sequence. Such libraries are used for detecting antibodies as well as for estimating their variety and specificity [105]. Synthetic peptide libraries also provide epitope mapping of protein antigens, the process of locating the epitope on the protein surface or in the protein sequence [106]. There are many peptide synthesis methods: biological (peptide is expressed on the surface of bacterium or phage) or fully synthetic (peptide is synthesized on the abiotic surface like cellulose or polypropylene) [107]. 

Determination of antigenic determinants of protein may be intricate considering the existence of discontinuous (assembled), apart from continuous (sequential, linear), epitopes [106]. However, several strategies of epitope mapping are available. The most often used strategy is array-based oligo-peptide scanning. This technique uses a library of oligo-peptide sequences from overlapping and non-overlapping segments of a target protein and tests for their ability to bind the antibody of interest. This method is fast and relatively inexpensive, and specifically suited to profile epitopes for a large number of candidate antibodies against defined targets [108].

**Fig. (8):**
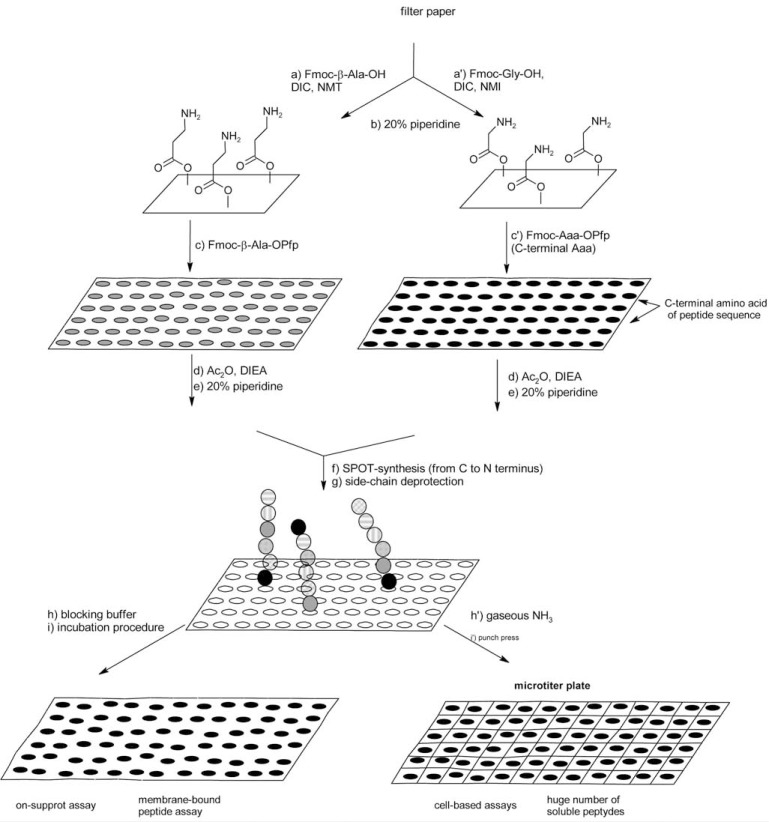
Scheme of SPOT synthesis.

So far the most general approach for epitope mapping has been developed by Ronald Frank [109]. The applied methodology, known as SPOT synthesis, is a special type of solid phase peptide synthesis proceeding directly on the membrane support, inside relatively small, separated spots regarded as separate reaction vessels. The method was initiated as an uncomplicated manual technique for parallel chemical synthesis of peptide arrays followed by an assay with appropriate interacting molecules performed directly on the membrane or in solution (Fig. **8**) [105]. 

SPOT method is useful for mapping not only linear, but also discontinuous epitopes as well as for characterizing antibodies [110, 111, 112]. It was even applied for identification of peptide mimicking the structure of an epitope (mimotope) [110]. A broad variety of other biomolecular binding events or enzymatic modifications can be investigated by using peptide arrays (prepared by the SPOT technique) such as protein-protein interactions [113-118] protein-DNA interactions [119] peptide-cell interactions [120-122] or enzyme-substrate interactions [123-127]. 

Synthesis of peptide on the membrane surface allows application of simple techniques, like dot-blot, for further investigations of e.g. bounded antibodies. However, the results obtained strongly depend on the structure of the linker fragment. The most classical linkers are prepared using 1-3 residues of β-alanine or glycine to separate peptide assembling with biomolecules used in assay out of the membrane [109, 128]. In SPOT technology, different linkers are also applied (like Carboxy-Frank-Linker, *p*-hydroxymethylbenzoic acid (HMB) linker, the Rink-amide linker or 4-hydroxymethyl-phenoxy acetic acid (HMPA) and 4-(4-hydroxymethyl-3-methoxyphenoxy)-butyric acid (HMPB) linkers) enabling cleavage of peptides from the support [129-133]. 

**Fig. (9):**
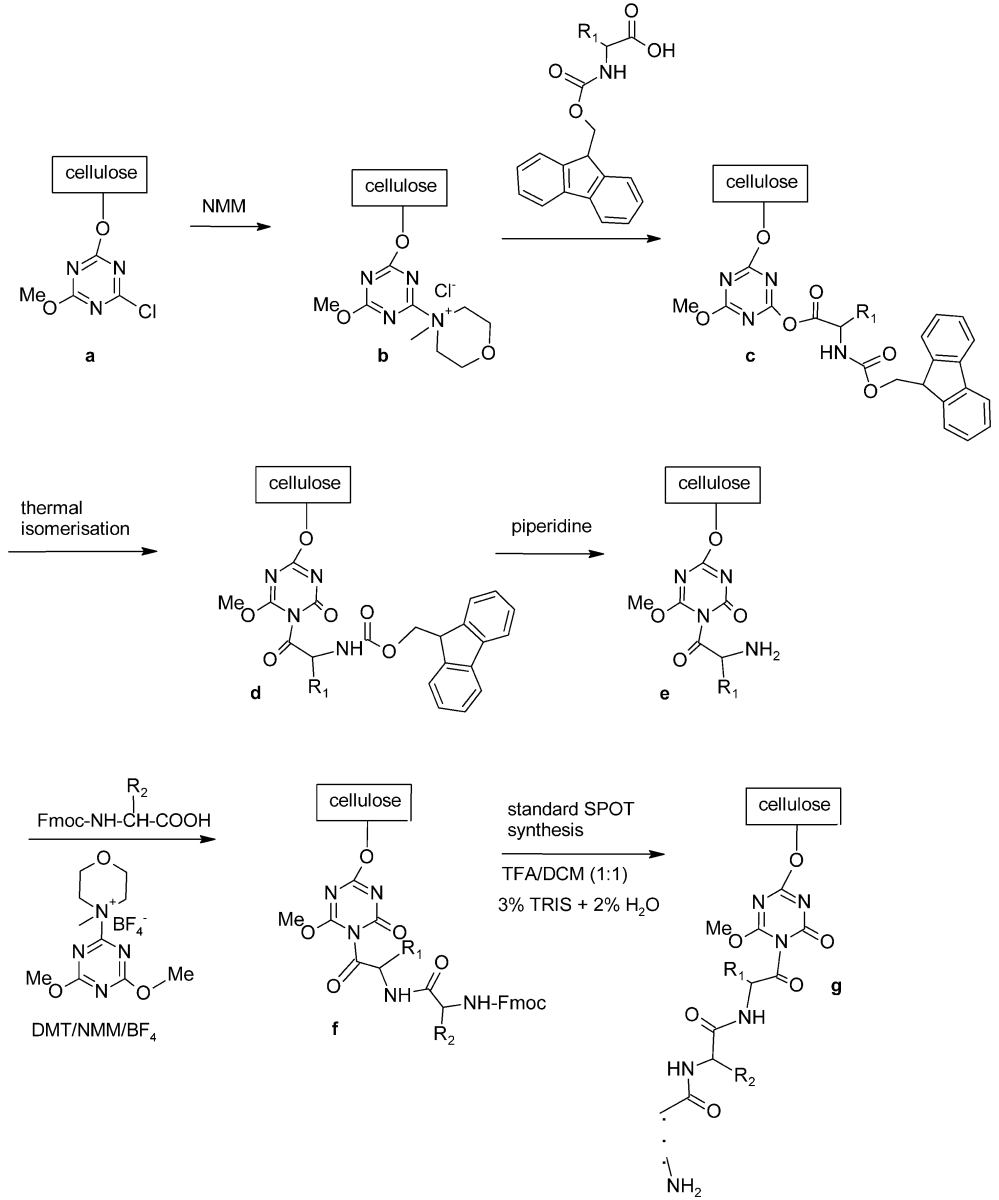
Synthesis of peptides with free N-termini anchored by isocyanuric linker.

A different type of anchoring the peptide chain to cellulose matrix was proposed by Kaminski and co-workers [6, 134-136]. 1-Acyl-3,5-dimethyl-1,3, 5-triazin-2,4,6 (1H,3H, 5H)-trion derivatives serve both as a spacer and a linker. This isocyanuric linker has been introduced by thermal isomerization of 2-acyloxy-4,6-dimethoxy-1,3,5-triazines already immobilized on the cellulose support [137]. A synthetic procedure leading to peptides anchored to cellulose by 1-acyl-3,5-dimethyl-1,3,5-triazin-2,4,6(1H,3H,5H)-trion (iso-MT) is shown in (Fig. **9**). In the first step, chloro-triazine immobilized on cellulose was treated with N-methylmorpholine yielding *N*-triazinylammonium chloride, which is activating the carboxylic function of Fmoc-protected amino acid yielding superactive triazine type ester [135, 136], which, in refluxing toluene, rearranges to a stable isocyanuric derivative. 

During peptide synthesis, anchoring method is essential for further reaction of peptide and antibody (Table **4**). 

For isocyanuric linker, interactions with antibodies were found more selective [138]. This linker was applied during synthesis of peptides corresponding to a flap region of different ureases (bacterial and plant) based on the flap region from *H. pylori* urease. Those peptides were useful in investigating human sera. It was possible to differentiate sera of patients with autoimmune diseases like rheumatoid arthritis or atherosclerosis from sera of healthy donors [6, 7]. On the other hand, a library of 361 peptides, where each peptide had a sequence with one substituted amino acids in a defined position, was applied to determine amino acids which are essential for antibody binding [139].

For epitope mapping of UreA and UreB subunits of *H. pylori* urease, Geysen’s method was adopted, which is based on the synthesis of the target amino acid sequence as a series of overlapping peptides on polypropylene pins [30]. 

Studies on urease epitopes were also performed using other techniques. One of the most interesting new developments in the search for novel antigens applied a computational method to predict T-cell epitopes using the whole genome sequence information [128]. 

In a more classical approach, antibody binding epitopes in *H. pylori* urease were determined with monoclonal antibodies produced in Balb/c mice. After digestion of bacterial urease with trypsin followed by separation of peptides with affinity chromatography, monoclonal antibodies were applied to identify epitopes. Next, amino acid sequences of isolated peptides were determined by mass spectroscopic analysis. Fujii *et al.* showed that two such peptides, with the sequence SVELIDIGGNRRIFGFNALVDR and IFGFNAL VDR, were recognized by two monoclonal antibodies (MAb): HpU-2 and HpU-18 respectively. Based on the data of competitive binding determined by using surface plasmon resonance and analysis of the epitope for HpU-2 and HpU-18, it has been found that both MAb recognize almost the same position on the UreA subunit of *H. pylori* urease. An unanticipated result was suppressing of urease activity *via* an allosteric effect, which might cause a distortion of the conformation of the enzyme. On the other hand, the second mechanism observed in the case of several MAb studied (which in fact possessed a weaker inhibitory effect, such as HpU-17 and -20) is assumed that MAb B binds to the vicinity of the active site, resulting in the reduction of the urease activity [140].

Molecular biology methods are also used in *H. pylori* urease epitope determination. Nineteen truncated fragments of gene coding UreB subunit were amplified and cloned into the prokaryotic expression vector pET-28a (+) or pGEX-4T-2. After verification, the constructs obtained were transformed into *Escherichia coli* which expressed recombinant proteins. Using three MAbs against UreB of *H. pylori* (A1H10, A3C10, and B3D9) three linear B-cell epitopes, probably useful as the targets for development of epitope-based vaccines against *H. pylori*, were identified. These epitopes were localized in the aa regions: 158-172 (GGGTGPADGTNATTI), 181-195 (WMLRAAEEYS MNLGF), and 349-363 (TLHDMGIFSITSSDS) of UreB [141].

Urease is highly expressed by all strains of *H. pylori* and is immunogenic. Additionally this enzyme could stimulate generation of antibodies able to inhibit its activity. For this reason, it seems to be a promising vaccine target. However, vaccination of urease may not give a sufficient protective effect. A combination of urease with other antigens may yield better results. It seems that a fusion of UreB urease subunit with truncated HpaA surface protein may give a better protection than either protein alone [142]. 

There were also attempts to design a vaccine using mucosal adjuvant cholera toxin B subunit (CTB) and an epitope (UreA 183-203) of *H. pylori* urease. Both peptides were bound with the linker (DPRVPSS) to avoid the formation of new epitopes. The CTB-UreA epitope vaccine had good immunogenicity and immunoreactivity and induced specific neutralizing antibodies which showed an effectively inhibitory effect on *H. pylori* urease enzymatic activity [143].

Today, experiments to identify and characterize linear antibody epitopes using peptide scans, amino acids scans, substitutional analyses, truncation libraries, deletion libraries, cyclization scans, all types of combinatorial libraries and randomly generated libraries of single peptides are standard techniques widely applied even in non-specialized laboratories [135]. 

## CONCLUSIONS 

Urease is an enzyme studied for a long time. Its structure, synthesis and biochemical activity are known. There are also many studies concerning urease toxic effect on human tissues. However, its role in long-lasting autoimmune diseases is still controversial. Nevertheless, the presence of molecular mimicry between bacterial ureases and human proteins has been suggested [7, 101]. Proteins containing motives, similar to infectious agents, may function as autoantigens. In described autoantigenes, some similarities to ureases may be found [90, 101]. It was proved that this enzyme stimulates antibodies synthesis [89], but determination of epitopes in urease protein may be difficult and non-conclusive. Therefore investigations applying synthetic peptides could be very helpful in mapping epitopes both in infectious agents proteins as well as in determining amino acids located in epitopes which are essential for human humoral response [139]. Urease, although investigated for a long time, still seems to be an unexplored enzyme.

## Figures and Tables

**Table 1.** The Sequence Similarity of Structural and Accessory Polypeptides of Ureases of Different Bacteria.

Polypeptides	Identical amino acid sequence [%]				Reference

*Bordetella bronchiseptica*	*Alcaligenes eutrophus*	*K. aerogenes*		*P. mirabilis*	

γ (UreA)	84	79		73	[49]
β (UreB)	63	69		67	
α (UreC)	70	69		68	

UreD	43	33		28	
UreG	75	66		59	
UreE	39	38		38	
UreF	54	31		31	

*Actinobacillus pleuropneumoniae*	*Haemophilus influenzae*	*Bacillus* sp. TB-90	*H. pylori*	*K. aerogenes*	

γ (UreA)	96	67	64	57	[50]
β (UreB)	86	55	53	60	
α (UreC)	87	62	62	66	

UreE	85	21	28	25	
UreF	67	34	34	27	
UreG	95	60	66	60	
UreD	70*	24	29*	21	

* data for UreH

**Table 2.** Characteristic of Methods for Ureolytic Activity Determination.

Method	Description	Advantages	Disadvantages	Application	Reference

Qualitative					

Urea-phenol red-agar plate	activity is detected on the microbiological medium containing urea and phenol red as pH indicator. Bacteria alkalize medium by hydrolysis of urea, causing change of its color.	facility of realization inexpensive	restricted to cultivable bacteria able to grow on this medium results need multiplication of bacteria	suitable for routine detection of activity, not recommended for kinetic analysis	[53]
					[63]

Native gel electrophoresis	pH-dependent method. Sample containing urease is electrophoresed in native agarose or acrylamide gel. Active protein is detected after incubation of gel in solution containing urea and phenol red.	allows estimation of the size of active protein inexpensive	equipment for electrophoresis is indispensable time consuming		[64]
					[65]

Quantitative					

Phenol – hypochlorite assay	spectrophotometric method based on detection of ammonia released during urea hydrolysis. Ammonia reacts with phenol-hypochlorite at high pH forming indophenol	simple able to detect even a small amount of ammonia (<0.02 µmol)	requires numerous sampling of the reaction mixture sensitive to various factors like temperature and time, pH of buffers, inhibitors limited linearity of the calibration plots	very useful in full kinetic analyses, the most frequently used in scientific research	[8]
					[66]
					[67]
					[68]

Nesslerization reaction	spectrophotometric assay with Nessler reagent in colored pH indicator solution	easy to perform	needs titration with diluted HCl to determine ammonia amount long reaction time less sensitive than phenol-hypochlorite assay		[8]
					[69]

Coupled enzyme assay	spectrophotometric method based on coupling reaction of ammonia with α-ketoglutarate in presence of glutamate dehydrogenase (GLDH)	sensitive alternatively, a horseradish peroxidase may be used for ammonia detection	GLDH has pH optimum higher than most ureases sensitive to inhibitors difficult interpretation expensive		[70]
					[71]
					[72]

Potentiometric assays	method of direct monitoring of ammonia ions with ion-selective electrodes or ammonia-selective electrode	unaffected by inhibitors fast in performance allow continuous monitoring of activity	interference by potassium and other monovalent ions low sensitive (in ion-selective electrode) during assay, an ionic strength of solution changes (there is no buffer)	useful in determination of the urease inhibition mechanisms	[73]
					[74]

Isotopic methods	methods based on the urea with carbon isotope radioactive C^14^ or non-radioactive C^13^ (there are also methods based on N^15^). A isotope-labeled CO_2_ is detected	fast in performance	need scintillation counter (for C^14^) or mass spectrometer (for C^13^ and N^15^)	useful in diagnosis of *H. pylori* gastric mucosa infection	[75]
					[76]

**Table 3.** Pathologic Effect of Bacterial Ureases in Human Diseases.

Role of urease	Bacterium species	Disease	Reference
Surviving in host’s organism	*H. pylori*	gastritis, peptic ulcers	[1, 4, 80]
	*M. tuberculosis*	tuberculosis	[81]
	*E. coli*	hemorrhagic colitis, HUS	[82]
Persistence to host’s cells	*H. pylori*	gastritis, peptic ulcers	[29]
Precipitation of polyvalent ions	*P. mirabilis*,* M. morganii, U. urealyticum* and others	urinary tract infections	[1, 22, 24, 83]
Stimulation of inflammatory reaction	*H. pylori*	gastritis, peptic ulcers	[4]
	*Y. enterocolitica*	reactive arthritis	[1, 84]
Cytotoxic effect on host’s cells	*H. pylori*	gastritis, peptic ulcers	[1, 80]
Damage to glycosaminoglycan layer	*P. mirabilis*	urinary tract infections	[5]
Damage of tight junctions	*H. pylori*	peptic ulcers	[85]
Aggregation of blood platelets	*H. pylori*	gastritis, cardiovascular disease	[86]

HUS - hemolytic uremic syndrome

**Table 4.** Interaction of Antibodies with *H. pylori* Urease Epitopes with Free *N*-Termini Anchored on Cellulose.

Epitope	Peptide sequence	Reaction^a^	Specificity
	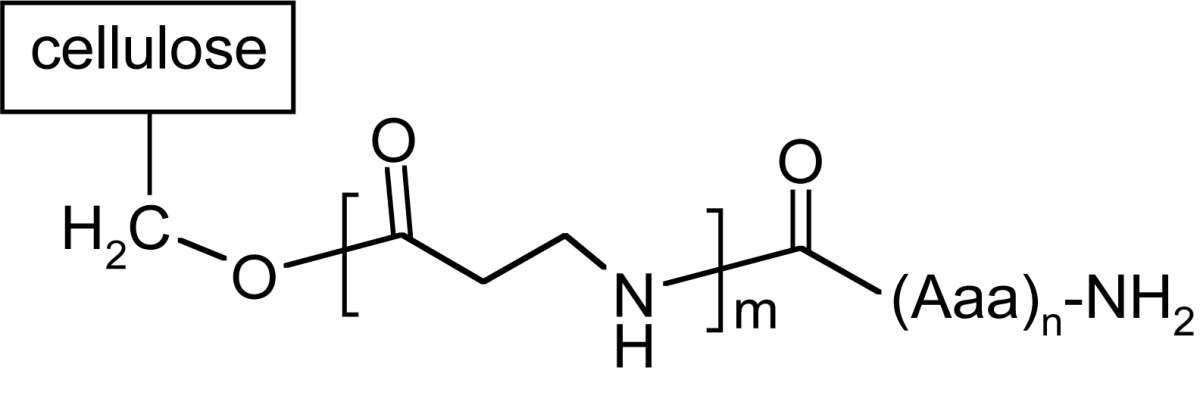		
UB-33	H_2_N-CHHLDKSIKEDVQFADSRI-COO-cellulose	-	0%
	H_2_N-CHHLDKSIKEDVQFADSRI-β-Ala-COO-cellulose	-	
	H_2_N-CHHLDKSIKEDVQFADSRI-β-Ala-β-Ala-β-Ala-COO-cellulose	-	
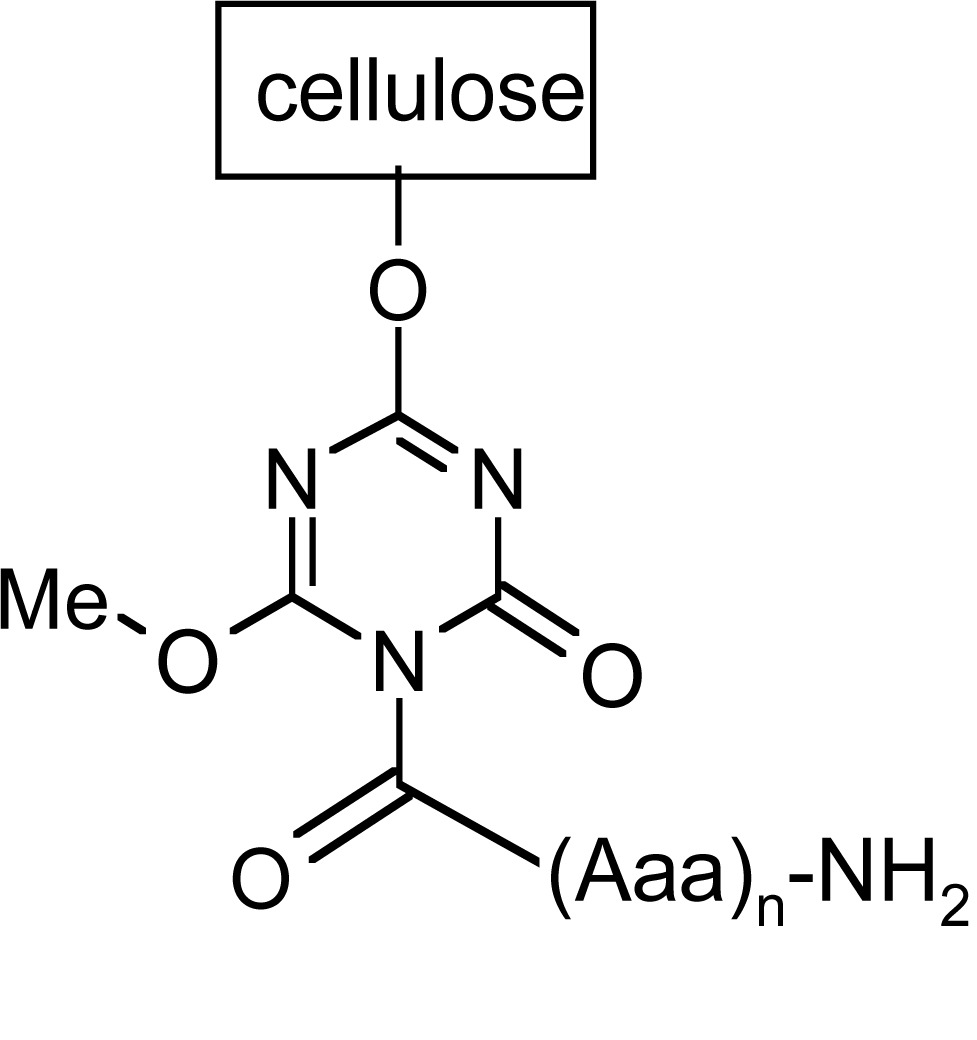			
UB-33	H_2_N-CHHLDKSIKEDVQFADSRI-β-Ala-iso-MT-cellulose	+, m	100%
	H_2_N-CHHLDKSIKEDVQFADSRI-iso-MT-cellulose	+, s	
F-8	H_2_N-SIKEDVQF-β-Ala-iso-MT-cellulose	+, s	
	H_2_N-SIKEDVQF-iso-MT-cellulose	+, m	

a (no reaction)

+ (reaction); s (strong); m (medium).

## References

[1] Mobley HLT, Island MD, Hausinger RP (1995). Molecular biology of microbial ureases. Microbiol. Rev.

[2] Newsholme E, Leech A (2011). Functional Biochemistry in Health and
Disease.

[3] Sirko A, Brodzik R (2000). Plant ureases: Roles and regulation. Acta Biochim. Pol.

[4] Dunn BE, Phadnis SH (1998). Structure, Function and Localization of *Helicobacter pylori* Urease. Yale J. Biol. Med.

[5] Follmer C (2010). Ureases as a target for the treatment of gastric and urinary infections. J. Clin. Pathol.

[6] Arabski M, Konieczna I, Sołowiej D, Rogoń A, Kolesińska B, Kamiński Z, Kaca W (2010). Are anti-*H. pylori* urease antibodies involved
in atherosclerotic disease?. Clin. Biochem.

[7] Konieczna I, Kwinkowski M, Kolesińska B, Kamiński Z, Frączyk J, Żarnowiec P, Kaca W (2012). Detection of Antibodies against Synthetic Peptides Mimicking Ureases Fragments in Sera of Rheumatoid Arthritis Patients. Prot. Pept. Lett.

[8] Mobley HLT, Hausinger RP (1989). Microbial ureases: significance, regulation, and molecular characterization. Microbiol. Rev.

[9] Suzuki K, Benno Y, Mitsuoka T, Takebe S, Kobashi K, Hase J (1979). Urease-Producing Species of Intestinal Anaerobes and Their Activities. Appl. Environ. Microbiol.

[10] Murchan S, Aucken HM, O’Neill GL, Ganner M, Cookson BD (2004). Emergence, Spread, and Characterization of Phage Variants of Epidemic Methicillin-Resistant *Staphylococcus aureus* 16 in England and Wales. J. Clin. Microbiol.

[11] Jin M, Rosario W, Watler E, Calhoun DH (2004). Development of a large-scale HPLC-based purification for the urease from *Staphylococcus leei* and determination of subunit structure. Protein Expr. Purif.

[12] Clemens DL, Lee B-Y, Horwitz MA (1995). Purification, Characterization, and Genetic Analysis of *Mycobacterium tuberculosis* Urease, a Potentially Critical Determinant of Host-Pathogen Interaction. J. Bacteriol.

[13] Dupuy B, Daube G, Popoff MR, Cole ST (1997). *Clostridium perfringens* Urease Genes Are Plasmid Borne. Infect. Immun.

[14] Lam S, Yeo M (1980). Urease-Positive *Vibrio parahaemolyticus* Strain. J. Clinical Microbiol.

[15] Futagami S, Takahashi H, Norose Y, Kobayashi M (1998). Systemic and local immune responses against *Helicobacter pylori* urease in patients with chronic gastritis: distinct IgA and IgG productive sites. Gut.

[16] Nakano M, Iida T, Ohnishi M, Kurokawa K, Takahashi A, Tsukamoto T, Yasunaga T, Hayashi T, Honda T (2001). Association of the Urease Gene with Enterohemorrhagic *Escherichia coli* Strains Irrespective of Their Serogroups. J. Clin. Microbiol.

[17] Orth D, Grif K, Dierich MP, Würzner R (2006). Prevalence, structure and expression of urease genes in Shiga toxin-producing *Escherichia coli* from humans and the environment. Int. J. Hyg. Environ.-
Health.

[18] Tange Y, Niwa O (1997). Identification of the *ure1*+ gene encoding urease in fission yeast. Curr. Genet.

[19] Yu JJ, Smithson SL, Thomas PW, Kirkland TN, Cole GT (1997). Isolation and characterization of the urease gene (URE) from the pathogenic fungus *Coccidioides immitis*. Gene.

[20] Zonia LE, Stebbins NE, Polacco JC (1995). Essential role of urease in germination of nitrogen-limited *Arabidopsis thaliana* seeds. Plant Physiol.

[21] Pedrozo HA, Schwartz Z, Luther M, Dean DD, Boyan BD, Wiederhold ML (1996). A mechanism of adaptation to hypergravity in
the statocyst of *Aplysia californica*. Hear Res.

[22] Hedelin H (2002). Uropathogens and urinary tract concretion formation and catheter encrustations. Int. J. Antimicrob. Agents.

[23] Carter EL, Proshlyakov DA, Hausinger RP (2011). Apoprotein isolation and activation, and vibrational structure of the *Helicobacter mustelae* iron urease. J. Inorg. Biochem.

[24] Rózalski A, Kwil I, Torzewska A, Baranowska M, Staczek P (2007). *Proteus* bacilli: features and virulence factors. Postepy Hig. Med. Dosw.

[25] Lv J, Jiang Y, Yu Q, Lu S (2011). Structural and functional role of nickel ions in urease by molecular dynamics simulation. J Biol Inorg Chem.

[26] Mörsdorf G, Kaltwasser H (1990). Cloning of the genes encoding urease from *Proteus vulgaris* and sequencing of the structural genes. FEMS Microbiol. Lett.

[27] Walz SE, Wray SK, Hull SI, Hull RE (1988). Multiple proteins encoded within the urease gene complex of *Proteus mirabilis*. J. Bacteriol.

[28] Jones BD, Mobley HLT (1989). *Proteus mirabilis* urease: nucleotide sequence determination and comparison with jack bean urease. J. Bacteriol.

[29] Schoep TD, Fulurija A, Good F, Lu W, Himbeck RP, Schwan C, Choi S S, Berg DE, Mittl PRE, Benghezal M, Marshall BJ (2010). Surface Properties of *Helicobacter pylori* Urease Complex Are Essential for Persistence. PLoS ONE.

[30] Clayton CL, Pallen MJ, Kleanthous H, Wren BW, Tabaqchali S (1990). Nucleotide sequence of two genes from *Helicobacter pylori* encoding for urease subunits. Nucleic Acids Res.

[31] Ferrero RL, Labigne A (1993). Cloning, expression and sequencing of *Helicobacter felis* urease genes. Mol. Microbiol.

[32] Labigne A, Cussac V, Courcoux P (1991). Shuttle cloning and nucleotide sequences of *Helicobacter pylori* genes responsible for urease activity. J. Bacteriol.

[33] Solnick JV, O’Rourke J, Lee A, Tompkins LS (1994). Molecular analysis of urease genes from a newly identified uncultured species of *Helicobacter*. Infect. Immun.

[34] Lee SG, Calhoun DH (1997). Urease from a Potentially Pathogenic Coccoid Isolate: Purification, Characterization, and Comparison to Other Microbial Ureases. Infect. Immun.

[35] Lee MH, Mulrooney SB, Renner MJ, Markowicz Y, Hausinger RP (1992). *Klebsiella aerogenes* urease gene cluster: sequence of *ureD* and demonstration that four accessory genes (*ureD*, *ureE*, *ureF*, *ureG*) are involved in nickel metallocenter biosynthesis. J. Bacteriol.

[36] Lee MH, Pankratz HS, Wang S, Scott RA, Finnegan MG, Johnson MK, Ippolito JA, Christianson DW, Hausinger RP (1993). Purification and characterization of *Klebsiella aerogenes* UreE protein: a nickel-binding protein that functions in urease metallocenter assembly. Protein Sci.

[37] Mulrooney SB, Hausinger RP (1990). Sequence of the *Klebsiella aerogenes* urease genes and evidence for accessory proteins facilitating nickel incorporation. J. Bacteriol.

[38] Park I-S, Carr MB, Hausinger RP (1994). *In vitro* activation of urease apoprotein and role of UreD as a chaperone required for nickel metallocenter assembly. Proc. Natl. Acad. Sci. USA.

[39] Park I-S, Hausinger RP (1995). Evidence for the presence of urease apoprotein complexes containing UreD, UreF, and UreG in cells that are competent for *in vivo* enzyme activation. J. Bacteriol.

[40] Sriwanthana B, Island MD, Maneval D, Mobley HLT (1994). Single step purification of *Proteus mirabilis* urease accessory protein UreE, a protein with a naturally occurring histidine tail, by nickel chelate affinity chromatography. J. Bacteriol.

[41] Sriwanthana B, Island MD, Mobley HLT (1993). Sequence of the *Proteus mirabilis* urease accessory gene *ureG*. Gene.

[42] Voland P, Weeks DL, Marcus EA, Prinz C, Sachs G, Scott D (2003). Interactions among the seven *Helicobacter pylori* proteins encoded by the urease gene cluster. Am. J. Physiol. Gastrointest. Liver Physiol.

[43] Park I-S, Hausinger RP (1995). Requirement of CO_2_ for *in vitro* assembly of the urease nickel metallocenter. Science.

[44] Musiani F, Zambelli B, Stola M, Ciurli S (2004). Nickel trafficking: insights into the fold and function of UreE, a urease metallochaperone. J. Inorg. Biochem.

[45] Palinska KA, Jahns T, Rippka R, Tandeau de Marsac N (2000). *Prochlorococcus marinus* strain PCC 9511, a picoplanktonic cyanobacterium, synthesizes the smallest urease. Microbiology.

[46] Ha N-C, Oh S-T, Sung JY, Cha KA, Lee MH, Oh B-H (2001). Supramolecular assembly and acid resistance of *Helicobacter pylori* urease. Nat. Struct. Biol.

[47] McMillan DJ, Mau M, Walker MJ (1998). Characterisation of the urease gene cluster in *Bordetella bronchiseptica*. Gene.

[48] Bosse JT, MacInnes JI (1997). Genetic and Biochemical Analyses of *Actinobacillus pleuropneumoniae* Urease. Infect. Immun.

[49] Monteiro L, de Mascarel A, Sarrasqueta AM, Bergey B, Barberis C, Talby P, Roux D, Shouler L, Goldfain D, Lamouliatte H, Megraud F (2001). Diagnosis of *Helicobacter pylori* infection: noninvasive methods compared to invasive methods and evaluation of two new tests. Am. J. Gastroenterol.

[50] Falsafi T, Favaedi R, Mahjoub F, Najafi M (2009). Application of Stool-PCR test for diagnosis of *Helicobacter pylori* infection in children. World J. Gastroenterol.

[51] Ragsdale SW (2009). Nickel-based Enzyme Systems. J. Biol. Chem.

[52] Konieczna I (2010). Characteristic of the bacterial ureases molecular
variety and estimation of reactivity of the human anti-urease antibodies. PhD Thesis.

[53] Hamilton-Miller JMT, Gargan RA (1979). Rapid screening for urease inhibitors. Invest. Urol.

[54] Christensen WB (1946). Urea decomposition as a means of differentiating *Proteus* and paracolon cultures from each other and from *Salmonella* and *Shigella* types. J. Bacteriol.

[55] Hussain Qadri SM, Zubairi S, Hawley HP, Mazlaghani HH, Ramirez EG (1984). Rapid test for determination of urea hydrolysis. Antonie van Leeuwenhoek.

[56] Bell GD, Weil J, Harrison G, Morden A, Jones PH, Gant PW, Trowell JE, Yoong AK, Daneshmend TK, Logan RFA (1987). 14C-urea breath analysis, a non-invasive test for *Campylobacter pylori* in the stomach. Lancet.

[57] Vakil N, Rhew D, Soll A, Ofman JJ (2000). The costeffectiveness of diagnostic testing strategies for *Helicobacter pylori*. Am. J. Gastroenterol.

[58] Mansour-Ghanaei F, Sanaei O, Joukar F (2011). Clinical Validation of
an Office-Based 14C-UBT (Heliprobe) for *H. pylori* Diagnosis in Iranian Dyspeptic Patients. Gastroenterol. Res. Pract.

[59] Foroutan M, Loloei B, Irvani S, Azargashb E (2010). Accuracy of rapid urease test in diagnosing *Helicobacter pylori* infection in patients using NSAIDs. Saudi. J. Gastroenterol.

[60] Krogfelt KA, Lehours P, Megraud F (2005). Diagnosis of *Helicobacter pylori* Infection. Helicobacter.

[61] Artiko VM, Obradović VB, Petrović NS, Davidović BM, Grujić-Adanja GS, Nastić-Mirić DR, Milosavljević TN (2001). 14C-urea breath test in the detection of *Helicobacter pylori* infection. Nuc. Med. Rev.

[62] Jassal MS, Nedeltchev GG, Lee J-H, Choi SW, Atudorei V, Sharp ZD, Deretic V, Timmins GS, Bishai WR (2010). ^13^[C]-Urea Breath Test as a Novel Point-of-Care Biomarker for Tuberculosis Treatment and Diagnosis. PLoS ONE.

[63] Ruiz-Herrera J, Gonzalez J (1969). A continuous method for the measurement of urease activity. Anal. Biochem.

[64] Blattler DP, Contaxis CC, Reithel FJ (1967). Dissociation of urease by glycol and glycerol. Nature.

[65] Shaik-M MB, Guy AL, Pancholy SK (1980). An improved method for the detection and preservation of urease activity in polyacrylamide gel. Anal. Biochem.

[66] Weatherburn MW (1967). Phenol-hypochlorite reaction for determination of ammonia. Anal. Chem.

[67] Krajewska B, Chudy M, Drozdek M, Brzozka Z (2003). Potentiometric
Study of Urease Kinetics over pH 5.36 -8.21. Electroanalysis.

[68] Juszkiewicz A, Kot M, Zaborska W (1998). Calorimetric study of
inhibition of urease by 2-mercaptoethanol. Procedures based upon
integrated rate equations. Thermochim. Acta.

[69] Lin Y-L, Chen Ch-T, Lin S-C, Lee C, Kuo H-S, Shih C-M, Hsu Y-H, Chin Y-P, Chan E-C (2000). A simple method to determine urea concentration using intact *Helicobacter pylori* and Bromo Cresol Purple as a pH indicator. Biotechnol. Lett.

[70] Kaltwasser H, Schlegel HG (1966). NADH-dependent coupled assay for urease and other ammonia-producing systems. Anal. Biochem.

[71] Gambhir A, Gerard M, Mulchandani AK, Malhotra BD (2001). Coimmobilization of Urease and Glutamate Dehydrogenase in Electrochemically Prepared Polypyrrole-Polyvinyl Sulfonate Films. Appl. Biochem. Biotechnol.

[72] Stutts P, Fridovich I (1964). A continual spectrophotometric determination of ammonia-producing systems. Anal. Biochem.

[73] Katz SA (1964). Direct potentiometric determination of urease activity. Anal. Chem.

[74] Montalvo JC (1970). An improved urease electrode. Anal. Biochem.

[75] McDonald JA, Speeg KV, Campbell JW (1972). Urease: a sensitive and specific radiometric assay. Enzymologia.

[76] Wrong OM, Vince AJ, Waterlow JC (1985). The contribution of endogenous urea to face alammonia in man, determined by 15N-labeling of plasma urea. Clin. Sci.

[77] Solomon CM, Alexander JA, Glibert PM (2007). Measuring urease activity in aquatic environmental samples. Limnol. Oceanogr. Methods.

[78] Karmali K, Karmali A, Teixeira A, Curto MJ (2004). The use of Fourier transform infrared spectroscopy to assay for urease from *Pseudomonas aeruginosa* and *Canavalia ensiformis*. Anal. Biochem.

[79] Kumar S, Barth A (2010). Following Enzyme Activity with Infrared Spectroscopy. Sensors.

[80] Olivera-Severo D, Wassermann1 GE, Carlini CR (2006). Ureases
display biological effects independent of enzymatic activity. Is
there a connection to diseases caused by urease-producing bacteria?. Braz. J. Med. Biol. Res.

[81] Lin W, Mathys V, Ang EL, Koh VH, Martínez Gómez JM, Ang ML, Zainul Rahim SZ, Tan MP, Pethe K, Alonso S (2012). Urease activity represents an alternative pathway for *Mycobacterium tuberculosis* nitrogen metabolism. Infect. Immun.

[82] Steyert SR, Kaper JB (2012). Contribution of Urease to Colonization by Shiga Toxin-Producing *Escherichia coli*. Infect. Immun.

[83] Coker C, Poore CA, Li X, Mobley HLT (2000). Pathogenesis of *Proteus mirabilis* urinary tract infection. Microbes Infect.

[84] Probst P, Hermann E, Meyer Zum Buschenfelde K-H, Fleischer B (1993). Identification of the *Yersinia enterocolitica* Urease β Subunit as a Target Antigen for Human Synovial T Lymphocytes in Reactive Arthritis. Infect. Immun.

[85] Wroblewski LE, Shen L, Ogden S, Romero-Gallo J, Lapierre LA, Israel DA, Turner JR, Peek RM (2009). *Helicobacter pylori* dysregulation of gastric epithelial tight junctions by urease-mediated myosin II activation. Gastroenterology.

[86] Wassermann GE, Olivera-Severo D, Uberti AF, Carlini CR (2010). *Helicobacter pylori* urease activates blood platelets through a lipoxygenase-mediated pathway. J. Cell. Mol. Med.

[87] Johnson DE, Russell RG, Lockatell CV, Zulty JC, Warren JW, Mobley HLT (1993). Contribution of *Proteus mirabilis* Urease to Persistence, Urolithiasis, and Acute Pyelonephritis in a Mouse Model of Ascending Urinary Tract Infection. Infect. Immun.

[88] Burne RA, Chen Yi-YM (2000). Bacterial ureases in infectious diseases. Microbes Infect.

[89] Hirota K, Nagata K, Norose Y, Futagami S, Nakagawa Y, Senpuku H, Kobayashi M, Takahashi H (2001). Identification of an antigenic epitope in *Helicobacter pylori* urease that induces neutralizing antibody production. Infect. Immun.

[90] Backes C, Ludwig N, Leidinger P, Harz C, Hoffmann J, Keller A, Meese E, Lenhof H-P (2011). Immunogenicity of autoantigens. BMC Genom.

[91] Thomas JE, Whatmore AM, Barer MR, Eastham EJ, Kehoe MA (1990). Serodiagnosis of *Helicobacter pylori* Infection in Childhood. J. Clin. Microbiol.

[92] Dunn BE, Cohen H, Blaser MJ (1997). Helicobacter pylori. Clin. Microbiol. Rev.

[93] Leal-Herrera Y, Torres J, Perez-Perez G, Gomez A, Monath T, Tapia-Conyer R, Muñoz O (1999). Serologic IgG Response To Urease In *Helicobacter pylori*-Infected Persons From Mexico. Am. J. Trop. Med. Hyg.

[94] Nurgalieva ZZ, Conner ME, Opekun AR, Zheng CQ, Elliott SN, Ernst PB, Osato M, Estes MK, Graham DY (2005). B-Cell and T-Cell Immune Responses to Experimental *Helicobacter pylori* Infection in Humans. Infect. Immun.

[95] Burnie JP, Al-Dughaym A (1996). The application of epitope mapping in the development of a new serological test for *Helicobacter pylori* infection. J. Immunol. Methods.

[96] Milioti N, Bermudez-Fajardo A, Penichet ML, Ernesto O-O (2008). Antigen-Induced Immunomodulation in the Pathogenesis of Atherosclerosis. Clin. Dev. Immunol.

[97] Oshima T, Ozono R, Yano Y, Oishi Y, Teragawa H, Higashi Y, Yoshizumi M, Kambe M (2005). Association of *Helicobacter pylori* Infection With Systemic Inflammation and Endothelial Dysfunction in Healthy Male Subjects. J. Am. Coll. Cardiol.

[98] Aletaha D, Neogi T, Silman AJ, Funovits J, Felson DT, Bingham CO, Birnbaum NS, Burmester GR, Bykerk VP, Cohen MD, Combe B, Costenbader KH, Dougados M, Emery P, Ferraccioli G, Hazes JMW, Hobbs K, Huizinga TWJ, Kavanaugh A, Kay J, Kvien TK, Laing T, Mease P, Menard HA, Moreland LW, Naden RL, Pincus T, Smolen JS, Stanislawska-Biernat E, Symmons D, Tak PP, Upchurch KS, Vencovský J, Wolfe F, Hawker G (2010). 2010 Rheumatoid Arthritis Classification Criteria. Arthritis & Rheumatism.

[99] Rashid T, Ebringer A (2007). Rheumatoid arthritis is linked to *Proteus*—the evidence. Clin. Rheumatol.

[100] Tobón GJ, Youinou P, Saraux A (2010). The environment, geoepidemiology, and autoimmune disease: Rheumatoid arthritis. J. Autoimmun.

[101] Wilson C, Tiwana H, Ebringer A (2000). Molecular mimicry between HLA-DR alleles associated with rheumatoid arthritis and *Proteus mirabilis* as the aetiological basis for autoimmunity. Microbes Infect.

[102] Appel H, Mertz A, Distler A, Sieper J, Braun J (1999). The 19 kDa protein of *Yersinia enterocolitica* O:3 is recognized on the cellular and humoral level by patients with *Yersinia* induced reactive arthritis. J. Rheumatol.

[103] Murphy TF, Brauer AL (2011). Expression of urease by *Haemophilus influenzae* during human respiratory tract infection and role in survival in an acid environment. BMC Microbiol.

[104] Contreras-Rodriguez A, Quiroz-Limon Jose, Martins AM, Peralta H, Avila-Calderon E, Sriranganathan N, Boyle SM, Lopez-Merino A (2008). Enzymatic, immunological and phylogenetic characterization of *Brucella suis* urease. BMC Microbiol.

[105] Shin D-S, Kim D-H, Chung W-J, Lee Y-S (2005). Combinatorial Solid Phase Peptide Synthesis and Bioassays. J. Biochem. Mol. Biol.

[106] Benjamin DC, Berzofsky JA, East IJ, Gurd FR, Hannum C, Leach SJ, Margoliash E, Michael JG, Miller A, Prager EM, Reichlin M, Sercarz EE, Smith-Gill SJ, Todd PE, Wilson AC (1984). The antigenic structure of proteins: a reappraisal. Annu. Rev. Immunol.

[107] Liu R, Enstrom AM, Lam KS (2003). Combinatorial peptide library methods for immunobiology research. Exp. Hematol.

[108] O’Brien-Simpson NM, Pathirana RD, Paolini RA, Chen Y-Y, Veith PD, Tam V, Ally N, Pike RN, Reynolds EC (2005). An Immune Response Directed to Proteinase and Adhesin Functional Epitopes Protects against *Porphyromonas gingivalis*-Induced Periodontal Bone Loss. J. Immunol.

[109] Frank R (1992). Spot-synthesis: an easy technique for the positionally addressable, parallel chemical synthesis on a membrane support. Tetrahedron.

[110] Mahler M, Fritzler MJ (2010). Epitope specificity and significance in systemic autoimmune diseases. Ann. N.Y. Acad. Sci.

[111] Schwemmle M, Billich C (2004). The use of peptide arrays for the characterization of monospecific antibody repertoires from polyclonal sera of psychiatric patients suspected of infection by Borna disease virus. Mol. Divers.

[112] Hilpert K, Hansen G, Wessner H, Küttner G, Welfle K, Seifert M, Höhne W (2001). Anti-c-myc antibody 9E10: epitope key positions and variability characterized using peptide spot synthesis on cellulose. Protein Eng.

[113] Tong J, Elowe S, Nash P, Pawson T (2003). Manipulation of EphB2 regulatory motifs and SH2 binding sites switches MAPK signaling and biological activity. J. Biol. Chem.

[114] Yaffe MB, Rittinger K, Volinia S, Caron PR, Aitken A, Leffers H, Gamblin SJ, Smerdon SJ, Cantley LC (1997). The structural basis for 14-3-3: phosphopeptide binding specificity. Cell.

[115] Nash P, Tang X, Orlicky S, Chen Q, Gertler FB, Mendenhall MD, Sicheri F, Pawson T, Tyers M (2001). Multisite phosphorylation of a CDK inhibitor sets a threshold for the onset of DNA replication. Nature.

[116] Espanel X, Wälchli S, Rückle T, Harrenga A, Huguenin-Reggiani M, van Huijsduijnen RH (2003). Mapping of synergistic components of weakly interacting protein-protein motifs using arrays of paired peptides. J. Biol. Chem.

[117] Huang H, Li L, Wu C, Schibli D, Colwill K, Ma S, Li C, Roy P, Ho K, Songyang Z, Pawson T, Gao Y, Li SS-C (2008). Defining the specificity space of the human SRC homology 2 domain. Mol. Cell. Proteomics.

[118] Smith MJ, Hardy WR, Murphy JM, Jones N, Pawson T (2006). Screening for PTB domain binding partners and ligand specificity using proteomederived NPXY peptide arrays. Mol. Cell Biol.

[119] Reuter M, Schneider-Mergener J, Kupper D, Meisel A, Mackeldanz P, Krüger DH, Schroeder C (1999). Regions of endonuclease *EcoRII* involved in DNA target recognition identified by membrane-bound peptide repertoires. J. Biol. Chem.

[120] Kato R, Kaga C, Kanie K, Kunimatsu M, Okochi M, Honda H (2011). Peptide Array-Based Peptide-Cell Interaction Analysis. Mini-Rev Org. Chem.

[121] Kato R, Kaga C, Kunimatsu M, Kobayashi T, Honda H (2006). Peptide arraybased interaction assay of solid-bound peptides and anchorage-dependant cells and its effectiveness in cell-adhesive peptide design. J. Biosci. Bioeng.

[122] Falsey JR, Renil M, Park S, Li S, Lam KS (2001). Peptide and small molecule microarray for high throughput cell adhesion and functional assays. Bioconjug. Chem.

[123] Thiele A, Pösel S, Spinka M, Zerweck J, Reimer U, Reineke U, Schutkowski M (2011). Profiling of Enzymatic Activities Using Peptide Arrays. Mini-Reviews in Organic Chemistry.

[124] Leung GC, Murphy JM, Briant D, Sicheri F (2009). Characterization of kinase target phosphorylation consensus motifs using peptide SPOT arrays. Methods Mol. Biol.

[125] Houseman BT, Huh JH, Kron SJ, Mrksich M (2002). Peptide chips for the quantitative evaluation of protein kinase activity. Nat. Biotechnol.

[126] Tegge WJ, Frank R (1998). Analysis of protein kinase substrate specificity by the use of peptide libraries on cellulose paper (SPOT-method). Methods Mol. Biol.

[127] Espanel X, Huguenin-Reggiani M, van Huijsduijnen RH (2002). The SPOT technique as a tool for studying protein tyrosine phosphatase substrate specificities. Protein Sci.

[128] Moss SF, Moise L, Lee DS, Kim W, Zhang S, Lee J, Rogers AB, Martin W, De Groot AS (2011). HelicoVax: epitope-based therapeutic *Helicobacter pylori* vaccination in a mouse model. Vaccine.

[129] Hoffmann S, Frank R (1994). A new safety-catch peptide-resin linkage for the direct release of peptides into aqueous buffers. Tetrahedron Lett.

[130] Panke G, Frank R (1998). Improved Preparation of a Safety-Catch Linker for the Solid Phase Synthesis of Peptide Acids Finally Released into Aqueous Buffers. Tetrahedron Lett.

[131] Scharn D, Wenschuh H, Reineke U, Schneider-Mergener J, Germeroth L (2000). Spatially addressed synthesis of amino- and amino-oxy-substituted 1, 3,5-triazine arrays on polymeric membranes. J. Comb. Chem.

[132] Rau HK, DeJonge N, Haehnel W (2000). Combinatorial Synthesis of Four-Helix Bundle Hemoproteins for Tuning of Cofactor Properties. Angew. Chem. Int. Ed. Engl.

[133] Ay B, Landgraf K, Streitz M, Fuhrmann S, Volkmer R, Boisguerin P (2008). Using hydroxymethylphenoxy derivates with the SPOT technology to generate peptides with authentic C-termini. Bioorg. Med. Chem. Lett.

[134] Kaca W, Kaminski ZJ, Kolesinska B, Kwinkowski M, Arabski M, Konieczna I (2009). Peptides mimicking urease, methods of manufacturing, application in diagnostic tests and the way of performance the test. Patent Applications PCT/PL2009/000106,
WO/2010/071462.

[135] Kamiński ZJ, Paneth P, Rudzinski J (1998). A study on the activation of carboxylic acids by means of 2-chloro-4,6-dimethoxy-1,3,5-triazine and 2-chloro-4,6-diphenoxy-1,3,5-triazine. J. Org. Chem.

[136] Kamiński ZJ, Paneth P, O'Leary M (1991). Nitrogen Isotope Effects on the Acylation of Aniline J. Org. Chem.

[137] Kamiński ZJ, Główka ML, Olczak A, Martynowski D (1996). Thermal
isomerization of 2- acyloxy-4,6-dimethoxy-1,3,5-triazines to 1-acyl-3,5-dimethyl-1,3,5-triazin-2,4,6(1*H*,3*H*,5*H*)-triones. Crystal structure of 1-(2,2-dimethylpropanoyloxy)3,5-dimethyl-1,3,5-triazin-2,4,6(1*H*,3*H*,5*H*)-trione. Pol. J. Chem.

[138] Kolesińska B, Grabowski S, Konieczna I, Kaca W, Peroni E, Papini AM, Rovero P, Kamiński ZJ, Rolka K, Rekowski P, Silberring J (2007). Peptides 2006.

[139] Glenska J, Adamus-Białek W, Kwinkowski M, Kolesińska B, Kamiński Z, Kaca W (2011). In: Abstracts of the VI Congress of Polish
Biotechnology “IV EUROBIOTECH 2011” and Central European
Congress of Live Science, Kraków, Poland, October 12-15, 2011. Acta Biochim. Pol.

[140] Fujii R, Morihara F, Oku T, Hifumi E, Uda T (2004). Epitope mapping and features of the epitope for monoclonal antibodies inhibiting enzymatic activity of *Helicobacter pylori* urease. Biotechnol. Bioeng.

[141] Qiua Y, Wanga Y-C, Taoa H-X, Zhana D-W, Yuana S-L, Wanga P, Wanga L-C, Hanb X-P, Li C-S, Li J-K, Liua C-J (2010). Identification of B-cell epitopes in urease B subunit of *Helicobacter pylori* bound by neutralizing antibodies. Vaccine.

[142] Flach CF, Svensson N, Blomquist M, Ekman A, Raghavan S, Holmgren J (2011). A truncated form of HpaA is a promising antigen for use in a vaccine against *Helicobacter pylori*. Vaccine.

[143] Guo L, Li X, Tang F, He Y, Xing Y, Deng X, Xi T (2012). Immunological features and the ability of inhibitory effects on enzymatic activity of an epitope vaccine composed of cholera toxin B subunit and B cell epitope from *Helicobacter pylori* urease A subunit. Appl. Microbiol. Biotechnol.

